# Structural and Functional Effects of Traditional Chuño Processing on Potato Starch (*Solanum* spp.)

**DOI:** 10.3390/foods15122180

**Published:** 2026-06-17

**Authors:** Fabiola Valdivieso, José Luis Vila, Patricia Mollinedo, Luis Apaza Ticona

**Affiliations:** 1 Chemistry Research Institute, Faculty of Pure and Natural Sciences, Universidad Mayor de San Andrés, Building, 2nd Floor, Laboratory 6, Calle 27, Cota Cota, Campus Universitario, Av. Andrés Bello, La Paz 10077, Bolivia; faavaldivieso@gmail.com (F.V.); jvila@fcpn.edu.bo (J.L.V.); pmollinedo@fcpn.edu.bo (P.M.); 2 Organic Chemistry Unit, Department of Chemistry in Pharmaceutical Sciences, Faculty of Pharmacy, University Complutense of Madrid, Plza. Ramón y Cajal s/n, 28040 Madrid, Spain

**Keywords:** chuño processing, potato starch, starch structural modification, amylose content, pasting properties, X-ray diffraction

## Abstract

Potato starch (*Solanum* spp.) undergoes structural and functional modifications during traditional Andean chuño production; however, the integrated effects of processing history, cultivar-associated characteristics, and field-based environmental conditions remain insufficiently characterised. This study investigated the effects of chuño processing on the compositional, pasting, morphological, molecular, and crystalline properties of starches isolated from three potato cultivars (*Condor Imilla*, *Luk’i Turno*, and *Dutch Désirée*). Native and chuño starches were characterised by amylose quantification, viscoamylography, scanning electron microscopy (SEM), Fourier-transform infrared spectroscopy (FT-IR), and X-ray diffraction (XRD), together with severe thermal treatment to evaluate structural stability. Chuño processing was associated with a reduction in amylose content across all cultivars (6.9–23.4%) and an increase in gelatinisation onset temperature of approximately 21.5% (from ~65 °C to ~79 °C). Peak viscosity decreased substantially after processing (457.5–1110 BU to 194.5–442.5 BU), while breakdown values remained close to zero, indicating increased resistance to viscosity loss during heating. SEM analysis revealed changes in granule morphology and size distribution associated with chuño processing and subsequent thermal treatment, with more pronounced reductions in granule size observed in *Condor Imilla* and *Luk’i Turno* than in *Dutch Désirée*. FT-IR analysis demonstrated modifications in short-range molecular organisation without the appearance of new functional groups, indicating structural reorganisation rather than chemical transformation. XRD analysis confirmed that all starches retained the native B-type crystalline polymorph after chuño processing, although reductions in diffraction intensity and peak definition indicated decreased long-range structural order. Severe thermal treatment eliminated detectable crystalline order in all samples, producing predominantly amorphous diffraction profiles. Overall, chuño processing was associated with reduced swelling capacity, lower paste viscosity, enhanced thermal stability, and multiscale structural reorganisation while preserving the fundamental B-type polymorph. Given that the plant material originated from distinct agroecological environments and that traditional chuño production involved a variable number of processing cycles, the observed differences should be interpreted as integrated responses of starch systems to processing history and material characteristics rather than strictly genotype-driven effects. These findings highlight the potential of chuño as a naturally modified starch system with distinctive technological properties.

## 1. Introduction

One of the most important tuber crops worldwide, potato (*Solanum tuberosum*) represents a major natural source of starch for both industrial applications and human consumption [1,2]. Global production reached approximately 383 million tonnes in 2023 [3], underscoring its continuing importance for food security and its role as a key raw material for starch-based industries [4]. In Bolivia alone, annual production exceeds 1,167,000 tonnes, reflecting the crop’s central contribution to national dietary patterns and nutritional security [5].

The Andean region constitutes the centre of origin and diversification of cultivated potatoes and harbours an exceptional diversity of native landraces that differ in tuber morphology, pigmentation, biochemical composition, and technological properties [6]. This diversity provides a valuable source of genetic resources for food innovation and functional ingredient development. However, it is important to recognise that the physicochemical characteristics of Andean potatoes are influenced not only by genotype but also by environmental and agroecological factors, including altitude, soil composition, climate, and local agronomic practices. Consequently, differences observed among varieties cultivated under field conditions should be interpreted as reflecting the combined effects of genetic background and environmental influences rather than purely genotype-driven variability. Because these factors cannot be completely separated under traditional cultivation systems, this limitation should also be considered when interpreting processing-induced differences among cultivars.

Among this remarkable diversity, more than fifty native varieties have been reported to contain between 9 and 35 g of carbohydrates per 100 g fresh weight, highlighting their potential as sources of structurally diverse starches and bioactive compounds with functional relevance [7]. In addition to carbohydrates, Andean potatoes constitute valuable sources of proteins, minerals, vitamins, dietary fibre, and antioxidant compounds, making them important not only from a nutritional perspective but also as complex biological matrices whose composition may influence technological behaviour during processing [8].

From a compositional standpoint, Andean potato tubers exhibit considerable variability in proximate composition and micronutrient content. Moisture contents generally range from 68–84%, proteins from 7–14% on a dry basis, and carbohydrates from 79–87%, whereas lipids (0.4–1.5%) and dietary fibre (approximately 3.5%) are present in lower amounts. Likewise, mineral composition varies substantially, with iron concentrations ranging from 1–8.5 mg/100 g, calcium from 13–38 mg/100 g, and zinc from 1.2–2.3 mg/100 g depending on genotype, botanical species, ecotype, and cultivation conditions. Native potatoes also contain considerable antioxidant activity (approximately 150–180 µmol TE/100 g) together with phenolic compounds such as chlorogenic acid (around 90 mg/100 g) and flavonoids including epicatechin (approximately 460 mg/100 g), further illustrating their biochemical complexity [8,9]. Although starch represents the major constituent of potato dry matter, these additional components contribute to the overall physicochemical behaviour of the tuber and may indirectly influence starch functionality during processing.

Within this agronomic and nutritional context, chuño represents one of the most distinctive traditional potato products of the Andean highlands. Derived from the Quechua term *ch’uñu*, chuño refers to a naturally preserved potato obtained through an ancestral processing system based on repeated environmental freeze–thaw events followed by dehydration under high-altitude conditions [9,10]. This preservation technology has been practised for centuries and enables long-term storage while maintaining substantial nutritional and functional value [11]. Consequently, chuño constitutes both an important traditional food and a unique example of naturally modified plant material generated through environmental rather than industrial processing.

Chuño production is intrinsically linked to the climatic conditions of the Andean Altiplano, typically located above 3800–5000 m above sea level, where repeated nocturnal freezing, intense daytime solar radiation, and low relative humidity (25–40%) create a natural freeze–thaw dehydration environment [8,12]. Rather than representing a simple drying process, chuño production involves a complex sequence of physical and environmental events in which repeated water phase transitions progressively restructure plant tissues at cellular and molecular scales.

Accordingly, chuño processing should be regarded as a multistep and multi-stressor system involving freeze–thaw cycling [11,13], mechanical compression [14], solar and ultraviolet radiation exposure [9], and prolonged dehydration acting in a temporally overlapping manner. During nocturnal freezing, intracellular ice crystal formation generates mechanical disruption of parenchymatous tissues, whereas subsequent thawing promotes water migration and solute redistribution. Repeated mechanical pressing further accelerates moisture removal and contributes to irreversible tissue collapse. Simultaneously, exposure to intense solar and ultraviolet radiation induces oxidative and photochemical stresses that may affect enzymatic activity and the stability of biological macromolecules, including starch granules and membrane systems [12,15]. These processes typically occur between June and July, when night-time temperatures fluctuate between approximately −1 and −16 °C while daytime temperatures may exceed 20 °C, thereby intensifying repeated freeze–thaw cycles and dehydration [12].

Unlike industrial freeze-drying (lyophilisation), which is performed under controlled vacuum conditions and relies primarily on sublimation, chuño production depends on uncontrolled environmental phase transitions occurring under natural climatic conditions [16,17,18,19]. Consequently, the structural modifications generated during chuño production cannot be considered equivalent to those produced by conventional industrial dehydration technologies.

Within traditional Andean food systems, two principal products are recognised: black chuño and white chuño (*tunta*). Black chuño is produced through repeated freeze–thaw cycles followed by solar exposure, resulting in progressive dehydration and characteristic dark pigmentation, and is widely consumed as a staple food. White chuño, in contrast, undergoes additional protection from sunlight together with prolonged soaking or washing in natural water bodies, often lasting several days or even weeks before further drying. These processing differences generate distinct physicochemical and sensory characteristics and are reflected in their commercial and cultural value, with white chuño generally regarded as the higher-quality product and frequently reserved for ceremonial or festive occasions [12,16].

The soaking stage involved in white chuño production also plays an important biochemical role, particularly in reducing glycoalkaloid concentrations in bitter and semi-domesticated potato species such as *Solanum ajanhuiri*, *Solanum juzepczukii*, and *Solanum curtilobum*. During prolonged immersion, these compounds are partially leached into the surrounding water, thereby improving food safety while simultaneously modifying mineral composition through diffusion-driven exchange processes [12]. Depending on regional practices, soaking may continue for 20–30 days in rivers, lakes, or constructed pits, leading to substantial changes in mineral profiles. For example, calcium concentrations may increase to approximately 120 mg/100 g, whereas potassium and magnesium are frequently reduced because of leaching [8,12]. These observations illustrate that traditional processing directly influences the final nutritional composition of chuño.

From a structural perspective, starch constitutes approximately 65–80% of potato dry matter and largely determines the physicochemical behaviour of both native potatoes and chuño products [20,21]. Native potato starch exhibits a semi-crystalline organisation composed of alternating crystalline and amorphous lamellae in which amylopectin double helices and amylose-rich domains collectively determine swelling behaviour, gelatinisation, retrogradation, and digestibility. Repeated freeze–thaw cycles and dehydration are expected to modify this hierarchical organisation by altering water distribution, hydrogen-bonding interactions, and amylose–amylopectin associations, thereby influencing both structural organisation and functional properties [22,23].

Previous studies have demonstrated that freeze-drying or isolated freeze–thaw treatments may affect starch architecture and functionality; however, chuño production represents a considerably more complex process in which environmental freezing, thawing, mechanical pressing, solar exposure, and dehydration act sequentially and simultaneously [24]. Consequently, the observed modifications should be interpreted as the cumulative outcome of multiple sequential and interacting processing stages rather than the consequence of any single unit operation.

At the molecular level, these processes may promote the formation of more compact and thermally stable starch networks, modify polymer mobility, alter hydration dynamics, and influence amylose–amylopectin interactions. Such reorganisations have been associated with changes in gelatinisation behaviour, viscoelastic properties, shear resistance, retrogradation, and potentially the formation of slowly digestible or resistant starch fractions [13,17,18,19]. Nevertheless, the mechanisms through which traditional chuño processing modifies starch organisation across different structural levels remain incompletely understood, particularly when considering the inherent variability associated with different potato varieties cultivated under distinct agroecological conditions.

Importantly, freeze–thaw cycling, mechanical pressing, solar and ultraviolet radiation, and dehydration each contribute through different but temporally overlapping mechanisms. Freeze–thaw cycling primarily induces intracellular ice formation and mechanical disruption of cellular compartments; mechanical pressing accelerates irreversible tissue collapse and water removal; whereas solar and ultraviolet exposure mainly promotes oxidative and photochemical processes affecting both membrane integrity and starch macromolecular stability. Therefore, the structural evolution of potato tissues during chuño production should be interpreted as the cumulative consequence of multiple interacting stresses rather than the effect of any single dominant factor.

Despite nutrient losses occurring during processing, chuño retains considerable nutritional and functional relevance. Protein contents decrease from approximately 7–14% in fresh tubers to 0.6–4.5% in chuño and 1.3–3.2% in white chuño, whereas carbohydrates become proportionally enriched, representing approximately 92–96% on a dry matter basis. Antioxidant capacity also remains detectable (approximately 150–180 µmol TE/100 g), indicating partial preservation of bioactive compounds and supporting the continued importance of chuño as a functionally relevant food matrix within Andean diets [8,13]. Likewise, calcium enrichment during processing further illustrates the complex interactions between processing conditions and nutrient redistribution.

In this context, the present study provides a comprehensive comparative characterisation of native potato starch and starch obtained after traditional chuño processing from three Andean potato varieties. Amylose content, pasting behaviour, morphology, short-range molecular organisation, and crystalline structure were evaluated using viscoamylography, scanning electron microscopy (SEM), Fourier-transform infrared spectroscopy (FT-IR), and X-ray diffraction (XRD), together with severe thermal treatment to assess structural stability. Rather than attempting to isolate genotype effects—which cannot be fully separated from environmental influences under the present field-based experimental design—this work aims to characterise how traditional multi-step chuño processing is associated with structural and functional modifications within different starch systems. By integrating compositional, morphological, molecular, and crystalline analyses, this study contributes to a better understanding of the multiscale physicochemical transformations occurring during ancestral Andean processing and provides insights into the potential application of chuño-derived starches as naturally modified functional ingredients for future food systems. This approach provides a framework for understanding how traditional multi-step processing influences starch organisation across hierarchical structural levels and the resulting functional properties.

## 2. Materials and Methods

### 2.1. Sample Preparation

Three potato varieties exhibiting contrasting morphological, culinary, and agronomic characteristics were selected: *Condor Imilla* (*Solanum tuberosum* ssp. *andigena*), *Luk’i Turno* (*Solanum × juzepczukii*), a bitter Andean cultivar traditionally used for chuño production, and *Dutch Désirée* (*Solanum tuberosum*). Tubers were collected from representative agroecological zones in Bolivia within their typical cultivation ranges: *Condor Imilla* from Ayopaya municipality (Cochabamba; 17°15′ S, 66°10′ W; 3600–3800 m a.s.l.), *Luk’i Turno* from the Aroma community (Ingavi Province, La Paz; 16°33′ S, 68°16′ W; 3800–4000 m a.s.l.), and *Dutch Désirée* from Quillacollo Province (Cochabamba; 17°22′ S, 66°10′ W; 2200–3000 m a.s.l.). Taxonomic identification was confirmed at the National Herbarium of Bolivia, where voucher specimens were deposited under accession numbers HNB-Pot-001/2026, HNB-Pot-002/2026, and HNB-Pot-003/2026, respectively. All tubers were harvested at physiological maturity and stored under controlled conditions (10–12 °C and 85–90% relative humidity) prior to processing.

The selected materials represent contrasting phenotypic and agronomic profiles relevant to starch structure and processing behaviour. Because plant material was obtained from geographically distinct cultivation areas under field conditions, the system reflects naturally occurring environmental variability. Accordingly, the study evaluates starch properties as a combined outcome of cultivar identity and local growing conditions.

A summary of morphological, culinary, and agronomic traits is provided in Table 1.

### 2.2. Chuño Preparation

Chuño was produced following a modified traditional Andean freeze–sun drying process consisting of sequential freezing, solar thawing, mechanical pressing, and final dehydration. Initially, potato tubers were individually weighed to determine initial mass (120 ± 15 g) and selected to ensure uniform size and absence of visible defects.

Samples were frozen at −4 to −6 °C for three days using a Panasonic MDF-U533 freezer (Panasonic, Osaka, Japan; 2021) under static conditions with controlled air circulation and without direct tuber contact. After freezing, samples were thawed under direct solar exposure for one day under typical highland environmental conditions (8–15 °C; 30–50% relative humidity), with 7–9 h of daily solar radiation during June–July, according to local meteorological records.

Frozen–thawed tubers were then subjected to mechanical pressing using a Labor HP-5 hydraulic press (Labor, Madrid, Spain; 2019) at 0.5 MPa for 5 min. This sequence (freezing–thawing–pressing) constituted one processing cycle.

Each sample underwent between three and six cycles. This range reflects the intrinsic variability of traditional chuño production, in which processing is not predefined but adjusted according to tuber size and dehydration behaviour until an adequate dehydrated state is reached. Consequently, the number of cycles is not an independent experimental factor but a material-dependent outcome of the processing procedure.

Accordingly, cycle number may partially covary with cultivar identity and cannot be considered statistically independent across samples. The experimental design was not intended to control or normalise processing intensity, but to reproduce authentic production conditions in which raw material characteristics and processing progression are inherently linked.

Following cyclic processing, an additional freezing–thawing–pressing step was applied, after which tubers were peeled and subjected to ambient sun-drying until constant weight was achieved. Constant weight was defined as three consecutive measurements with mass variation below 0.1% over 24 h.

The protocol reproduces the integrated operational sequence of traditional chuño production under Andean highland conditions. Because all processing steps occur as a coupled system in practice, their individual contributions (freeze–thaw, solar exposure, mechanical pressing, and dehydration) cannot be experimentally isolated. Therefore, the structural and physicochemical outcomes reported here represent the combined effect of the complete traditional processing pathway under field-representative conditions.

### 2.3. Starch Extraction

Starch was isolated from potato and chuño samples using a semi-industrial procedure adapted from Kim et al. [26], with modifications reported by Frost et al. [27] and further adjusted in the present study to improve laboratory-scale reproducibility. For potato samples, tubers were thoroughly washed, peeled and cut into approximately 1 cm cubes. The cut material was immediately immersed in a 0.1% (*w*/*w*) sodium bisulphite solution (NaHSO_3_, Merck, CAS No. 7631-90-5, St. Louis, MO, USA) prepared in distilled water (1:1, *w*/*v*) to minimise enzymatic browning during processing.

The pre-treated samples were homogenised at high speed for 2 min using a Waring blender (Waring, Torrington, CT, USA), and the resulting slurry was filtered through a 140 μm mesh sieve. The starch-rich filtrate (“starch milk”) was refrigerated at 4 °C for 4 h to facilitate sedimentation, after which the supernatant was decanted. The sediment was resuspended in cold distilled water (1:1, *w*/*v*), allowed to settle for 15 min, and decanted again. This washing step was repeated until the supernatant appeared visually clear in order to remove residual soluble compounds and non-starch material. As a final purification step, the starch sediment was washed with 95% ethanol (EtOH, 95%, *v*/*v*, Merck, CAS No. 64-17-5, St. Louis, MO, USA) at a ratio of 1:0.5 (*w*/*v*), decanted and air-dried at room temperature (25 ± 5 °C) until constant weight was reached.

For chuño samples, the same extraction and purification procedure was applied following an additional hydration pre-treatment. Because chuño is denser, drier and structurally more rigid than fresh tubers, samples were first immersed in distilled water (1:1, *w*/*w*) for 48 h to restore moisture content and facilitate tissue disruption during homogenisation. The hydrated chuño was then cut into approximately 1 cm cubes and transferred to distilled water (1:2, *w*/*w*) before homogenisation. All subsequent extraction, filtration, sedimentation, washing and ethanol purification steps were identical to those used for potato samples.

This modified protocol was designed to standardise extraction efficiency across materials with markedly different moisture content and texture while preserving the native characteristics of the starch fraction as far as possible. To improve comparability between samples, all extractions were carried out under identical processing conditions using the same settling times, solvent ratios and drying criteria.

### 2.4. Thermal Treatment

To simulate domestic cooking conditions, starch samples were dispersed in water at a 1:3 (*w*/*v*) ratio in sealed polyethylene bags and heated at 100 °C for 20 min in a Thermo Scientific Precision 2810 water bath (Thermo Scientific, Waltham, MA, USA, 2022). Samples were subsequently cooled to 30–40 °C, rapidly frozen in liquid nitrogen (Air Liquide, Paris, France, 2021), and freeze-dried for 48 h using a FreeZone 2.5 L lyophiliser (Labconco, Kansas City, MO, USA, 2020). The dried material was then milled using an IKA A11 Basic grinder (IKA, Staufen, Germany, 2021). This combined thermal–freeze-drying protocol was used to stabilise structural changes induced by gelatinisation and retrogradation, enabling subsequent comparative analyses.

### 2.5. Amylose Quantification

Amylose content was determined spectrophotometrically following the method described by Aristizábal et al. [28], as applied to potato and chuño starches by Huanca López [29], with minor modifications. The assay is based on the formation of a blue amylose–iodine complex, in which iodine molecules are incorporated into the helical structure of amylose, producing a measurable absorbance at 620 nm.

For sample preparation, 100 mg of starch were weighed into 100 mL volumetric flasks. Subsequently, 1 mL of 95% ethanol (EtOH, ≥99.9% (GC), Merck, CAS No. 64-17-5, St. Louis, MO, USA) and 9 mL of 1 N sodium hydroxide (NaOH, pellets, ≥97.0%, Merck, CAS No. 1310-73-2, St. Louis, MO, USA) were added to each flask. The flasks were sealed and kept at room temperature for 18–24 h to allow complete starch dispersion. After this period, the volume was adjusted to 100 mL with distilled water.

An external calibration curve was prepared using potato amylose and amylopectin standards (from potato starch, Merck, CAS No. 9037-22-3, St. Louis, MO, USA). Standard mixtures containing 0, 10, 25, 30 and 40% amylose were prepared. For colour development, 5 mL aliquots of each standard solution were transferred into 100 mL volumetric flasks containing 50 mL distilled water, followed by the addition of 1 mL of 1 N acetic acid (AcOH, ≥99.7%, Merck, CAS No. 64-19-7, St. Louis, MO, USA) and 2 mL of 2% iodine solution (I_2_, ≥99.8%, Merck, CAS No. 7553-56-2, St. Louis, MO, USA). The mixtures were thoroughly mixed and brought to volume with distilled water.

The reaction mixtures were kept in the dark for 20 min before absorbance was measured at 620 nm using a UV–Vis spectrophotometer (Biotek Instruments, Inc., Winooski, VT, USA). Amylose content in the starch samples was calculated from the external calibration curve and corrected for sample moisture content, which was determined separately using a Moisture balance (MAC 110/WH; RADWAG Wagi Elektroniczne, Radom, Poland). The final amylose values were expressed on a dry-matter basis as mean ± standard deviation. Differences in amylose content among varieties were assessed using one-way analysis of variance (ANOVA), with variety treated as the single fixed factor; statistical significance was set at *p* < 0.05.

### 2.6. Pasting Properties

Pasting properties were evaluated using a Brabender Micro Visco-Amylo-Graph (model 21.1; Brabender, Duisburg, Germany, 2021), a standard instrument for starch pasting analysis. Moisture content was determined using a Radwag MAC 110/WH moisture analyser (Radwag, Radom, Poland, 2022). Starch suspensions were prepared at 5 g per 100 mL of distilled water (Milli-Q system, Merck Millipore, Burlington, MA, USA) and subjected to a controlled temperature programme consisting of heating from 30 to 80 °C at 7 °C/min, holding at 80 °C for 7 min 20 s, cooling to 50 °C at 8 °C/min, and final stabilisation at 50 °C. Breakdown viscosity (peak minus trough viscosity) and setback viscosity (final minus trough viscosity) were calculated from the resulting viscoamylograms in order to evaluate paste stability and retrogradation tendency.

### 2.7. Granule Morphology and Size

Starch granule morphology was analysed using optical microscopy and scanning electron microscopy (SEM, JEOL JSM-6610LV, JEOL Ltd., Tokyo, Japan). SEM imaging was performed with a Philips XL 30 microscope (Philips, Eindhoven, The Netherlands; 30 kV; 2021). For quantitative analysis, each micrograph was divided into 40 equal sections, from which 18 fields were randomly selected for measurement of maximum granule diameter using ImageJ software (NIH, Bethesda, MD, USA, version 1.53). This cluster-based sampling strategy provided representative measurements with 95% confidence and an approximate 3% margin of error.

### 2.8. Fourier Transform Infrared (FT-IR) Spectroscopy

FT-IR spectroscopy was used to evaluate structural differences among potato, chuño and thermally treated starch samples. Spectra were recorded using a Perkin Elmer 1000 FT-IR spectrometer (Perkin Elmer Inc., Waltham, MA, USA; 2000) equipped with a ZnSe universal attenuated total reflectance (ATR) accessory.

Starch samples were placed directly onto the ATR crystal and analysed without further preparation. Spectra were collected over the range of 4000–400 cm^−1^, and each spectrum represented the average of 16 consecutive scans. Background spectra were acquired prior to each measurement and automatically subtracted from the sample spectra.

The resulting spectra were processed using Spectrum v.3 software (Perking Elmer Inc., Shelton, CT, USA; version 2000). Characteristic absorption bands associated with hydroxyl groups, glycosidic linkages and starch molecular organisation were evaluated to identify structural modifications induced by chuño processing and thermal treatment. FT-IR analysis was employed as a complementary technique to provide information on short-range molecular order and structural changes in conjunction with X-ray diffraction analysis.

### 2.9. X-Ray Diffraction

Starch crystallinity was determined using an X-ray diffractometer (Rigaku Geigerflex; Rigaku, Tokyo, Japan; Cu Kα radiation, λ = 1.54 Å; scanning range 3–60° 2θ; 25 °C; 2020). Diffractograms were processed using X’Pert software (Malvern Panalytical, Malvern, UK, 2021) to identify crystalline patterns and quantify relative crystallinity, enabling comparison between varieties and processing conditions.

### 2.10. Statistical Analysis

Granule size data were analysed using SPSS v.28 (IBM Corp., Armonk, NY, USA). Normality was assessed using the Kolmogorov–Smirnov test, which indicated non-normal distributions across all datasets. Consequently, Kruskal–Wallis tests were applied to compare median granule diameters among varieties and processing conditions, followed by post hoc pairwise comparisons where appropriate. Statistical significance was set at *p* < 0.05, and results are reported as median values with interquartile ranges.

## 3. Results

### 3.1. Amylose Quantification of Potato and Chuño Starches

Table 2 summarises the amylose content of starch isolated from potato and chuño samples across the cultivars evaluated.

The amylose content of potato starch differed significantly among the three analysed varieties (one-way ANOVA, *p* = 0.001), with *Condor Imilla* exhibiting the highest value (36.89 ± 1.43%), followed by *Dutch Désirée* (32.60 ± 1.07%) and *Luk’i Turno* (30.78 ± 0.58%).

Given that plant material originated from geographically distinct agroecological zones and was not cultivated under a common-garden design, these differences should be interpreted as cultivar-associated variability under field-based conditions, with potential contributions from environmental factors such as altitude, soil composition, and local agronomic practices.

Amylose content in chuño starch also differed significantly among varieties (one-way ANOVA, *p* = 0.0003), ranging from 24.98 ± 0.73% in *Dutch Désirée* to 33.59 ± 0.90% in *Condor Imilla*. Although the relative ranking among cultivars was broadly maintained compared with native starches, the present experimental design does not allow separation of cultivar-associated variability, environmental background, and processing-related effects, since chuño production involved a variable number of processing cycles (three to six) determined by tuber characteristics rather than experimental standardisation.

A decrease in amylose content was observed after chuño processing in all varieties, although the magnitude and statistical significance varied. This reduction was statistically significant in *Condor Imilla* and *Dutch Désirée*, whereas *Luk’i Turno* exhibited a non-significant trend. Because processing intensity was not standardised and varied among samples, these observations are presented as integrated outcomes of material and processing history.

Several non-exclusive factors may contribute to the observed decrease, including differences in extraction efficiency between native and processed matrices, partial leaching of soluble components during repeated freeze–thaw and pressing cycles, and possible redistribution of amylose within the starch matrix. These interpretations remain tentative and should be considered as potential contributors rather than confirmed mechanisms.

Overall, while a consistent decrease in amylose content was observed in chuño starches, the present data do not allow this trend to be attributed to a single factor. The results more appropriately reflect the combined influence of processing history, starch extraction behaviour, and cultivar-associated characteristics under field-based conditions. Further studies using standardised processing protocols and controlled cultivation conditions would be required to disentangle these contributions.

### 3.2. Pasting Behaviour

**Figure 1:**
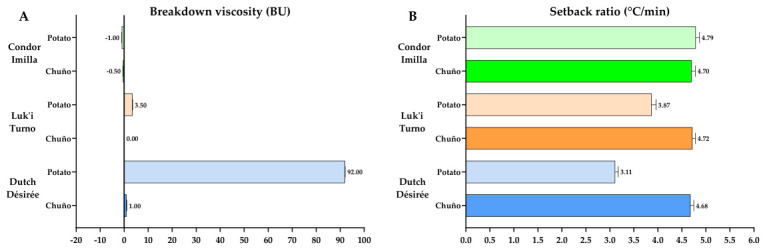
Breakdown viscosity (**A**) and setback ratio (**B**) of potato and chuño starch samples.

The pasting profiles of native potato and chuño starches revealed differences among the analysed samples, as summarised in Table 3 and Figure 1. Native starches displayed relatively similar onset gelatinisation temperatures across varieties, whereas chuño starches exhibited higher onset temperatures in all cases (Figures S1–S3). However, these differences should be interpreted cautiously, as they may reflect a combination of cultivar-associated characteristics, field-based environmental variability, and processing history rather than a single controlling factor.

More pronounced variability was evident in peak viscosity development. Among native starches, *Condor Imilla* exhibited the highest peak viscosity, whereas *Luk’i Turno* and *Dutch Désirée* presented lower values under the same analytical conditions. Although differences in viscosity magnitude were observed, the present experimental design does not allow these variations to be attributed exclusively to cultivar identity due to the absence of a common cultivation system and the lack of control over processing intensity in chuño samples.

This suggests that peak viscosity is influenced by combined sample-dependent factors, including intrinsic starch properties and structural modifications associated with field conditions and processing history, rather than a single determinant.

In all cases, chuño-derived starches exhibited lower peak viscosities compared with their corresponding native starches. However, because chuño preparation involved a variable number of freeze–thaw and pressing cycles (three to six), the extent to which this reduction is associated with processing severity, intrinsic cultivar characteristics, or differences in starch extraction behaviour cannot be independently resolved within the constraints of the present experimental design.

During cooling, all samples exhibited an increase in final viscosity relative to trough values, although the magnitude of this increase varied among cultivars and processing states. These differences may reflect variations in molecular-level reorganisation processes during cooling; however, the present analytical data do not allow identification of the specific structural factors responsible for these changes.

Breakdown and setback parameters further highlighted differences in paste stability among samples. Nevertheless, interpretation of these parameters should be made with caution, as they may be influenced by both intrinsic starch properties and prior sample history. Consequently, these parameters are best considered as comparative indicators of functional behaviour under identical analytical conditions rather than direct proxies of structural stability.

Overall, these findings indicate that pasting behaviour reflects an integrated response of starch properties and sample history under field-based conditions, rather than discrete cultivar-specific effects. The observed variations likely arise from combined differences in starch composition, granule behaviour during heating and cooling, and processing history inherent to non-standardised chuño preparation.

### 3.3. Morphological Characterisation of Starch Granules

#### 3.3.1. Effects of Thermal Treatment on Starch Granule Morphology

**Figure 2:**
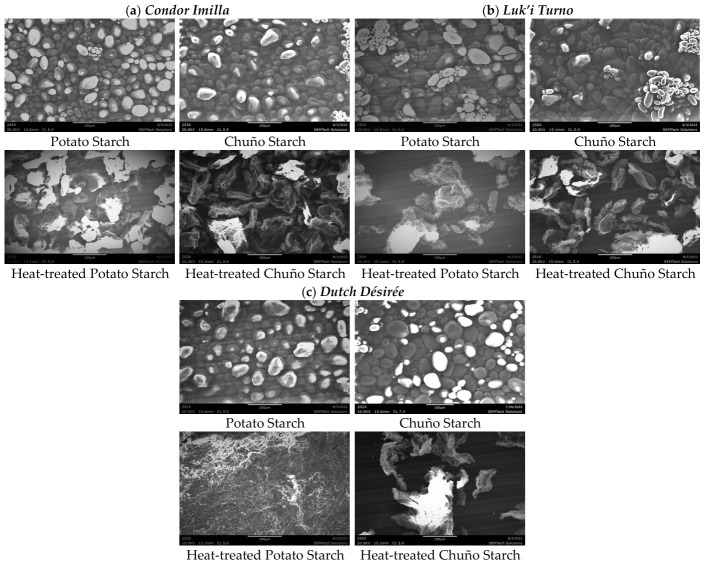
Scanning electron micrographs (SEM) of native and chuño starches from three potato varieties—(**a**) *Condor Imilla*, (**b**) *Luk’i Turno*, and (**c**) *Dutch Désirée*-before and after thermal treatment.

Scanning electron microscopy (SEM) analysis provided qualitative information on the morphological features of starch granules following thermal treatment (Figure 2 and Figures S7–S9). Heat treatment reduced moisture content to approximately 10–13%, corresponding to an average decrease of about 29% relative to native starches, and promoted granule aggregation was observed in which individual granules largely lost their original morphological identity. These observations should be interpreted as morphological outcomes under the specific experimental conditions applied, rather than as evidence of intrinsic structural transformation.

Both native and chuño-derived *Condor Imilla* samples exhibited broadly comparable aggregation patterns after thermal treatment and freeze-drying (Figures S4 and S7). However, this similarity likely arises from overlapping contributions of intrinsic starch properties, prior processing history, and the thermal–freeze-drying sequence applied, rather than a single governing factor.

Similarly, *Luk’i Turno* samples displayed differences between native and processed forms, with native starch forming more diffuse aggregates, whereas the processed sample exhibited comparatively more compact structures. These differences may be associated with variability in sample history, including the chuño processing sequence; however, the use of non-standardised processing (three to six freeze–thaw–pressing cycles depending on tuber characteristics) precludes disentangling the effects of processing severity from material-dependent behaviour.

Overall, while differences in aggregation behaviour were observed among samples, the present experimental design does not allow these effects to be attributed exclusively to cultivar identity, environmental background, or processing history. Accordingly, the observed morphological features should be considered as the integrated outcome of cultivar-associated characteristics under field conditions combined with the traditional chuño processing sequence.

#### 3.3.2. Granule Size Distribution

**Figure 3:**
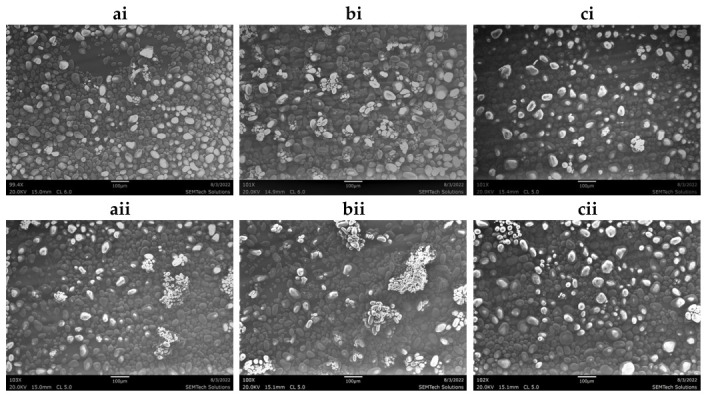
SEM micrographs at 100× magnification of native (**i**) and chuño (**ii**) starches from three potato varieties—(**a**) *Condor Imilla*, (**b**) *Luk’i Turno*, and (**c**) *Dutch Désirée*.

Quantitative analysis of granule diameters derived from SEM images (Table 4; Figure 3 and Figures S4–S6) revealed particle sizes ranging from approximately 15 to 75 µm, consistent with previously reported values for potato starch. Across all samples, the distributions were asymmetric, with noticeable differences in dispersion and shape between native potato starches and their corresponding chuño-derived starches.

*Dutch Désirée* chuño starch exhibited a relatively wider interquartile range (IQR), indicating higher variability within the central portion of the distribution. In contrast, *Luk’i Turno* and *Dutch Désirée* native starches exhibited narrower IQRs, indicating more concentrated distributions around the median. *Condor Imilla* chuño starch displayed pronounced positive skewness (skewness > 1.5) together with an expanded overall range, indicating a distribution influenced by a subset of larger granules. These differences are reported descriptively, as the present experimental design does not allow the underlying structural or processing-related determinants to be resolved.

The Kolmogorov–Smirnov test confirmed deviation from normality for all datasets; therefore, non-parametric Kruskal–Wallis tests were applied. No statistically significant differences (*p* > 0.05) were detected between *Condor Imilla* and *Luk’i Turno* in either native or chuño forms, nor between native and chuño starches of *Dutch Désirée*. Although non-significant trends in central tendency were observed in some comparisons, these must be interpreted with caution due to the absence of controlled cultivation conditions and standardised processing intensity.

The number of freeze–thaw–pressing cycles applied during chuño preparation (three to six cycles) may have influenced both granule integrity and the apparent distributional properties observed in SEM-derived measurements. Therefore, the reported size variations should be interpreted primarily as statistical descriptors of a heterogeneous population rather than as direct indicators of systematic changes in granule size driven by cultivar or processing effects.

Table 4 further highlights differences in distributional descriptors across samples. Across all groups, mean and median values indicate asymmetric distributions, while variance and standard deviation reflect differences in dispersion. *Luk’i Turno* potato starch exhibited a comparatively broader interquartile range, whereas *Condor Imilla* samples exhibited more concentrated central distributions.

Notably, *Condor Imilla* chuño starch displayed markedly elevated positive skewness (3.9), substantially higher than its native counterpart, indicating a distribution disproportionately influenced by a limited number of larger granules rather than a uniform shift in particle size. This observation is presented as a statistical descriptor of the dataset and does not imply a specific mechanistic interpretation.

### 3.4. Fourier Transform Infrared (FT-IR) Analysis

**Figure 4:**
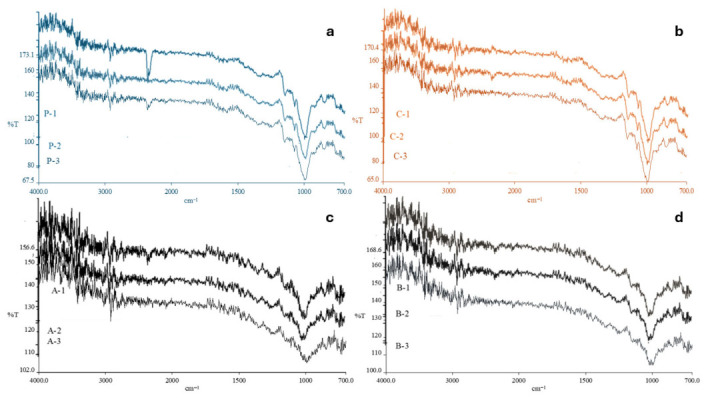
FT-IR spectra of native potato starch, chuño starch, and samples subjected to severe thermal treatment. Codes: P = native potato starch; C = chuño starch; A = native potato starch subjected to severe thermal treatment; B = chuño starch subjected to severe thermal treatment. (**a**) Spectra of native potato starch (*Condor Imilla*, *Luk’i Turno*, and *Dutch Désirée*; P-1 to P-3). (**b**) Spectra of chuño starch from the same varieties (*Condor Imilla*, *Luk’i Turno*, and *Dutch Désirée*; C-1 to C-3). (**c**) Spectra of native potato starch subjected to severe thermal treatment (*Condor Imilla*, *Luk’i Turno*, and *Dutch Désirée*; A-1 to A-3). (**d**) Spectra of chuño starch subjected to severe thermal treatment (*Condor Imilla*, *Luk’i Turno*, and *Dutch Désirée*; B-1 to B-3).

FT-IR spectra were collected from native potato starches (*Condor Imilla*, *Luk’i Turno*, and *Dutch Désirée*), their corresponding chuño-derived starches, and starches subjected to severe thermal treatment in both native and chuño forms (Figure 4).

All spectra were acquired under identical instrumental conditions and processed using a standardised workflow including baseline correction and vector normalisation to ensure inter-sample comparability. Each reported spectrum corresponds to the mean of three independent technical replicates (n = 3). Spectral reproducibility was verified across replicates prior to further analysis.

To enhance analytical robustness in the fingerprint region (1200–900 cm^−1^), second-derivative transformation was applied to resolve overlapping bands and confirm peak positions prior to quantitative evaluation. This approach reduces subjective peak assignment and improves reliability in complex polysaccharide systems.

All samples exhibited the characteristic FT-IR absorption profile of starch within the 4000–400 cm^−1^ region. The broad band at ~3300 cm^−1^ corresponds to O–H stretching vibrations associated with intra- and intermolecular hydrogen bonding, while weak absorptions at ~2920 cm^−1^ are attributed to C–H stretching vibrations of polysaccharide backbones.

The 1200–900 cm^−1^ region, dominated by C–O–C and C–O stretching vibrations of glycosidic linkages, was identified as the most sensitive spectral window for detecting variations in short-range molecular organisation. Spectral differences in band shape, intensity, and resolution across processing conditions were consistently observed across all replicates, indicating reproducible structural effects rather than experimental variability

**Figure 5:**
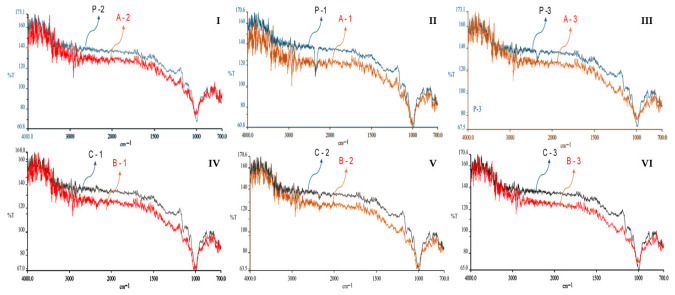
(**I**–**VI**) FT-IR spectra of native and thermally treated starch samples, presented as baseline-corrected and normalised spectra. The 1200–900 cm^−1^ region, associated with C–O–C and C–O stretching vibrations, exhibits variations consistent with differences in short-range molecular organisation among samples. Changes in band profile and intensity were also observed around approximately 1245 cm^−1^ and 1338 cm^−1^ following thermal treatment, which may reflect alterations in hydrogen-bonding interactions within the starch matrix.

Native and chuño starches from all cultivars exhibited relatively well-resolved spectral features in this region, indicating partial preservation of short-range molecular order. In contrast, severe thermal treatment induced systematic band broadening and reduced spectral resolution, consistent with increased molecular disorder. This effect was most pronounced in chuño-derived starches subjected to thermal treatment, which exhibited the lowest spectral definition across all samples (Figure 5).

Additional reproducible variations were observed at ~1370 cm^−1^ and ~1220 cm^−1^ in thermally treated samples, assigned to C–O–H bending and C–O stretching vibrations. These changes are consistent with perturbations in hydrogen-bonding interactions and increased local structural heterogeneity within the polysaccharide matrix.

In the 1000–1025 cm^−1^ region, native and chuño starches exhibited dominant bands near ~1047 cm^−1^ and ~1022 cm^−1^, associated with ordered and less ordered structural domains, respectively. Thermally treated samples displayed broader and partially overlapping features, particularly near ~996 and ~1004 cm^−1^, indicating reduced short-range molecular order and increased conformational heterogeneity.

No new absorption bands were detected in any sample, confirming that neither chuño processing nor thermal treatment induced detectable chemical modification within the sensitivity limits of FT-IR spectroscopy. Observed differences therefore arise from structural reorganisation of existing functional groups rather than chemical transformation.

To move beyond qualitative interpretation, short-range molecular order was quantified using two complementary FT-IR band intensity ratios derived from vector-normalised spectra: A_1047_/A_1022_ and A_1022_/A_995_. These ratios are widely accepted semi-quantitative descriptors of ordered (amylopectin double-helical) and less ordered (amorphous/amylose-rich) domains in starch systems. Peak separation and assignment were validated using second-derivative spectra, ensuring consistency across all replicates and experimental conditions.

Native starches consistently exhibited higher A_1047_/A_1022_ ratios compared with chuño-derived starches, indicating a higher proportion of short-range ordered domains. Among cultivars, *Dutch Désirée* exhibited the highest value (0.890 ± 0.008), indicating relatively higher native molecular organisation under field conditions (Table 5).

Following chuño processing, A_1047_/A_1022_ values decreased across all samples (e.g., *Dutch Désirée*: 0.670 ± 0.008), indicating a reproducible reduction in short-range molecular order. In parallel, the A_1022_/A_995_ ratio increased, providing a complementary and internally consistent indicator of increased amorphous contribution (Table 5).

Importantly, thermally treated samples exhibited smaller changes in both ratios compared with chuño-derived starches, indicating that the most relevant structural reorganisation occurs during freeze–thaw cycling, mechanical pressing, and dehydration rather than during subsequent thermal exposure. This distinction is supported by the convergence of FT-IR trends with XRD observations.

Overall, FT-IR results provide reproducible semi-quantitative evidence that chuño processing modifies the short-range molecular organisation of starch without inducing chemical changes. The consistency between band ratio analysis, second-derivative validation, and XRD-derived crystalline behaviour supports a coherent multiscale structural interpretation, in which chuño processing primarily alters the degree of molecular order rather than chemical composition or crystalline polymorph.

### 3.5. Crystalline Structure Analysis by X-Ray Diffraction

#### 3.5.1. Differences in Crystalline Structure Among Starch Samples

**Figure 6:**
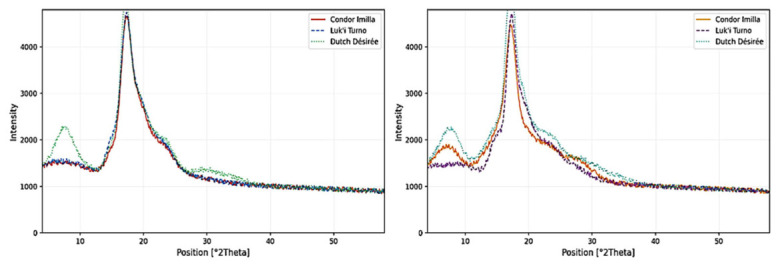
X-ray diffraction (XRD) patterns of native potato starches (**left**) and native chuño starches (**right**).

The B-type crystalline structure of native potato starch was confirmed by X-ray diffraction (XRD) analysis (Figure 6; Table S1). Characteristic diffraction peaks associated with B-type crystallinity are typically observed at 2θ values of approximately 5.6°, 15°, 17°, 22°, and 24° [30]. A more detailed diffraction fingerprint for potato starch has also been previously reported, including reflections across the approximate range of 5–34° 2θ [31]. These characteristic features were detected in all three potato varieties analysed, confirming the predominance of the B-type polymorph.

Despite sharing the same crystalline polymorph, differences in diffraction intensity and peak definition were observed among the potato starch samples under the present field-based conditions. *Dutch Désirée* exhibited relatively higher intensity at approximately 17.1°, which may reflect differences in diffraction intensity within the sensitivity limits of the technique, without allowing direct inference on crystalline order within the methodological sensitivity of XRD. However, since the plant material originated from distinct agroecological environments and was not grown under controlled conditions, these differences cannot be attributed exclusively to cultivar identity.

Minor variations in low- and high-angle reflections were also observed among samples, including a weak feature around approximately 7.5° and low-intensity reflections at higher angles in *Dutch Désirée*. These features are reported descriptively, as XRD does not allow unambiguous assignment of their structural origin in complex biological matrices. In contrast, *Luk’i Turno* and *Condor Imilla* exhibited broadly similar diffraction profiles, indicating comparable crystalline arrangements under the present experimental conditions.

The XRD patterns of chuño starches also corresponded to B-type crystallinity; however, differences in peak intensity and profile complexity were observed among samples. *Dutch Désirée* again exhibited comparatively higher intensity at approximately 17.1°, whereas *Luk’i Turno* and *Condor Imilla* exhibited lower intensities and more diffuse diffraction profiles. These variations reflect differences in diffraction profiles that cannot be uniquely attributed to cultivar, environmental conditions, or processing history within the present experimental design.

All chuño starch samples exhibited more complex diffraction profiles than their corresponding native starches, characterised by broader peaks and increased background scattering between approximately 14° and 30°. These features are consistent with a reduction in long-range crystalline order and an increased contribution of amorphous domains following chuño processing. However, XRD alone does not permit quantification of crystallinity changes with sufficient resolution to separate processing-induced effects from pre-existing structural variability.

Comparison between potato and chuño starches indicated that potato starches generally exhibited sharper diffraction peaks and higher peak intensities at equivalent 2θ positions, whereas chuño starches displayed broader and less resolved patterns. Although this trend is consistent across all samples, the variable number of freeze–thaw–pressing cycles applied during chuño preparation (three to six cycles depending on tuber characteristics) precludes direct attribution of these differences to processing severity alone.

Overall, potato starches exhibited more defined diffraction patterns, whereas chuño starches displayed broader and more heterogeneous profiles. These observations are consistent with structural modifications associated with freeze–thaw cycling, mechanical pressing, and dehydration during chuño processing. Nevertheless, because the cultivars originated from distinct agroecological environments and processing intensity was not standardised, the relative contribution of cultivar-associated characteristics, environmental conditions, and processing history cannot be independently resolved within the present experimental design.

#### 3.5.2. Thermally Induced Changes in Crystalline Structure and Their Structural Implications

**Figure 7:**
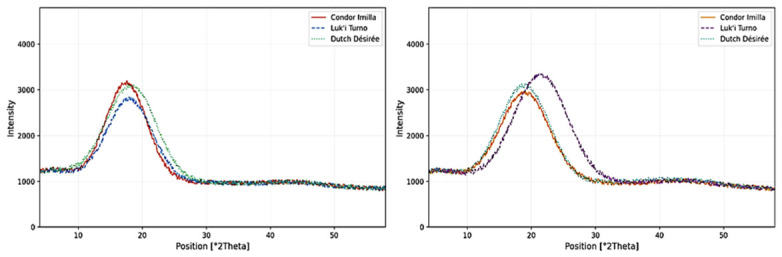
X-ray diffraction (XRD) patterns of thermally treated potato starches (**left**) and thermally treated chuño starches (**right**).

The X-ray diffraction patterns of the thermally treated potato starch samples exhibited the disappearance of the sharp reflection’s characteristic of the native B-type polymorph (Figure 7). These reflections were replaced by a broad diffuse halo centred at approximately 17.5–18.0°, consistent with extensive gelatinisation and the loss of long-range crystalline order associated with disruption of double-helical structures.

Following thermal treatment, all samples exhibited broadly similar amorphous diffraction profiles, indicating a comparable overall loss of crystalline order under the applied conditions. Minor variations in halo width and shape were observed among samples. In some cases, slightly broader or less defined halos were detected, whereas in others the profiles appeared relatively narrower. These differences are reported descriptively and may reflect variability in the extent of molecular rearrangement during gelatinisation; however, XRD alone does not allow further structural resolution of these effects.

**Figure 8:**
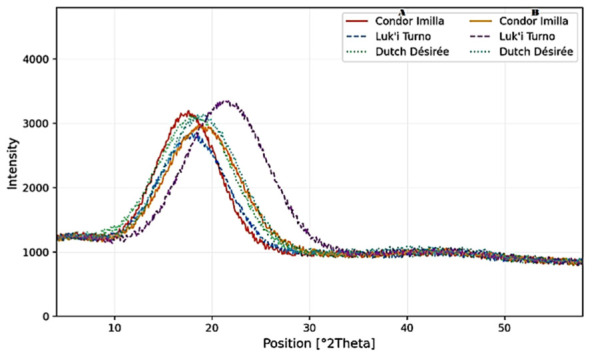
Comparative X-ray diffraction (XRD) patterns of thermally treated potato starches (**A**) and chuño starches (**B**).

Comparison between thermally treated potato and chuño starches revealed differences in the position and shape of the amorphous halo (Figure 8). Potato-derived starches exhibited a maximum centred at approximately 17.5–18.0°, whereas chuño-derived starches displayed broader halos extending towards higher angles (approximately 19–22°). These differences suggest subtle variations in the organisation of the amorphous starch matrix; however, the present data do not allow identification of the structural origin of these shifts.

Although long-range crystalline order was eliminated in all samples, differences in the resulting amorphous scattering profiles suggest that the thermal response may be influenced by prior processing history. Nevertheless, this interpretation remains tentative, as XRD provides limited information on molecular arrangement in amorphous systems, and the present experimental design does not allow separation of processing history effects from intrinsic sample variability.

In chuño-derived starches, the broader distribution of halo positions may indicate higher heterogeneity in the amorphous state. However, this observation is strictly descriptive and should not be interpreted as evidence of specific structural rearrangements.

Overall, thermal treatment resulted in complete loss of detectable crystalline order in all samples, while subtle differences in amorphous scattering profiles were observed. These findings are descriptive and reflect combined contributions from sample history and intrinsic variability, which cannot be independently resolved using the present experimental design.

## 4. Discussion

This study provides a comprehensive assessment of the structural and functional modifications induced by traditional chuño processing in potato starches from three Andean varieties (*Condor Imilla*, *Luk’i Turno*, and *Dutch Désirée*), highlighting differences associated with both cultivar identity and processing conditions. Chuño production is a multi-step traditional process involving environmental freezing, thawing cycles, mechanical pressing, and solar dehydration; therefore, the observed modifications arise from the combined and sequential action of these stages rather than a single unit operation.

In agreement with previous reports on chuño starch systems, freeze–thaw and dehydration sequences have been associated with increased gelatinisation temperatures (18–25%), attributed to strengthened intermolecular interactions during progressive structural reorganisation [18]. Consistently, the observed increase in onset gelatinisation temperature (~79 °C vs. ~65 °C in native starches) indicates enhanced granule thermal stability induced by chuño processing [32].

Compositional analysis revealed variability in amylose content among samples. *Luk’i Turno* and *Dutch Désirée* fall within the typical range for potato starch (20–30%) [33,34], whereas *Condor Imilla* exhibited higher values (37.12%), reflecting field-dependent variability associated with genotype–environment interactions during tuber development [35]. After chuño processing, a moderate reduction in amylose content (<10%) was observed across all samples, likely associated with partial leaching, solubilisation, and redistribution of amylose-rich fractions during repeated freeze–thaw cycles. Although extraction-related variability in partially reorganised starch matrices cannot be fully excluded, the consistency of the trend across cultivars supports a processing-associated effect. This reduction is expected to influence molecular reassociation during cooling due to the role of linear chains in hydrogen-bond-driven ordering processes [36,37].

These compositional changes are reflected in pasting behaviour, where an increase in thermal stability (~21.5%) was observed. This effect arises from cumulative structural rearrangements induced by freeze–thaw cycling, mechanical pressing, and dehydration, which collectively reduce water accessibility and restrict granule swelling. Although the mechanism is inherently multifactorial, it likely involves coupled changes in polymer mobility, hydration dynamics, and matrix densification [38,39].

FT-IR spectroscopy provided insights into short-range molecular organisation. In starch granules, crystallinity is governed by the ordered packing of amylopectin double helices, forming the semi-crystalline architecture of native starch [40]. All FT-IR spectra were acquired under identical conditions and analysed using baseline correction and vector normalisation and represent the mean of three independent measurements per sample, ensuring spectral reproducibility prior to ratio calculation. Second-derivative transformation was additionally applied to confirm peak positions in the fingerprint region (1200–900 cm^−1^), improving resolution of overlapping bands and increasing robustness of spectral assignment.

Across all samples, systematic variations in band shape and intensity in regions associated with C–O and C–O–C vibrations reflected changes in molecular order rather than chemical modification. A clear hierarchy of structural disruption was observed in the 1245 and 1338 cm^−1^ regions. Thermally treated samples exhibited pronounced band broadening and increased absorbance, indicating disruption of hydrogen-bonding networks and partial collapse of macromolecular organisation, as previously reported for thermally degraded polysaccharide systems [41,42]. In contrast, chuño-derived starches retained comparatively better-defined spectral features, indicating partial preservation of short-range order despite multi-step physical processing.

This hierarchical response was also evident in the fingerprint region (1200–900 cm^−1^), where native starches exhibited sharper spectral envelopes, chuño starches exhibited moderate broadening, and thermally treated samples displayed the highest loss of spectral resolution. These observations indicate that thermal treatment primarily amplifies pre-existing disorder, whereas chuño processing induces a structured reorganisation of starch architecture through repeated phase transitions and mechanical stress [43,44].

Semi-quantitative FT-IR analysis further strengthened these interpretations. The A_1047_/A_1022_ ratio (ordered domains) was highest in native starch (e.g., *Dutch Désirée*: 0.890 ± 0.008) and decreased after chuño processing (0.670 ± 0.008), indicating a reduction in short-range molecular order. Conversely, the A_1022_/A_995_ ratio increased, confirming a shift towards more disordered domains. The consistency of these trends across cultivars and replicates supports the robustness of the spectral metrics used for structural interpretation. Importantly, these FT-IR-derived changes are in agreement with XRD results, which indicate a reduction in long-range crystalline order while preserving B-type polymorphism, supporting a coherent multiscale structural model.

Crucially, the magnitude of change in FT-IR ratios was higher in chuño-derived samples than in thermally treated starches, indicating that freeze–thaw cycling, mechanical pressing, and dehydration collectively induce more extensive reorganisation than thermal exposure alone. This does not imply chemical transformation, but rather a deeper reconfiguration of hierarchical starch organisation across multiple structural levels.

X-ray diffraction confirmed that all samples retained B-type crystallinity, indicating that chuño processing does not alter the fundamental polymorphic structure. However, reduced peak intensity and sharpness revealed a decrease in crystalline order. The agreement between FT-IR (short-range order) and XRD (long-range order) supports a multiscale framework in which chuño processing progressively reduces structural organisation without inducing crystalline phase transformation [45,46,47].

Thermal treatment further highlighted differences in structural stability among cultivars. *Condor Imilla* exhibited the highest loss of diffraction intensity, indicating higher susceptibility to structural collapse, whereas *Luk’i Turno* retained higher residual order. *Dutch Désirée* exhibited intermediate stability. These differences are consistent with pasting behaviour, where more ordered systems exhibit higher resistance to thermal breakdown [48].

From a functional perspective, chuño starches exhibit properties comparable to physically modified starch systems, including moderate viscosity (~200–450 BU), reduced swelling capacity, and increased tendency towards molecular reassociation during cooling [49]. Setback values (~20% increase relative to native starches) further confirm enhanced chain reassociation, particularly in chuño starches (4.5–5%), whereas *Dutch Désirée* exhibited lower values, indicating reduced retrogradation tendency. These behaviours are consistent with increased formation of ordered associations upon cooling, potentially contributing to slowly digestible or resistant starch fractions [50,51].

The observed differences among samples reflect combined and inseparable contributions of cultivar identity, environmental growth conditions, and processing history. Given the absence of controlled cultivation conditions, these differences should be interpreted as associative rather than strictly genotype-driven and represent the integrated response of starch systems to both intrinsic and processing-related factors.

A critical factor is that chuño processing is defined by an endpoint rather than a fixed number of cycles, resulting in variable processing intensity among samples [52]. Therefore, structural and functional differences reflect the integrated outcome of raw material properties and processing severity. This inherent coupling precludes complete decoupling of cultivar effects from processing trajectory within the present experimental design.

The present study is limited by the absence of detailed molecular characterisation, including amylopectin fine structure, phosphate distribution, molecular weight profiles, and DSC thermal transitions. Additionally, the lack of enzymatic digestibility assays limits direct validation of inferred functional changes. Future studies incorporating these techniques would strengthen mechanistic interpretation of structure–function relationships.

Finally, while starch is the primary determinant of functionality, potato tubers also contain proteins and antioxidant compounds that may indirectly influence starch behaviour during processing. Incorporating these factors would enable a more holistic understanding of chuño-induced transformations.

## 5. Conclusions

Traditional chuño processing is associated with reproducible modifications in the structural and functional properties of potato starch from *Condor Imilla*, *Luk’i Turno*, and *Dutch Désirée*. These modifications arise from the combined and sequential effects of environmental freeze–thaw cycles, mechanical pressing, and solar dehydration, which collectively act on starch systems under non-standardised field conditions.

The present study indicates that starches from different cultivars exhibit inherent differences in their baseline structural and crystalline features. However, these intrinsic characteristics, together with environmental growing conditions, were treated as contextual background rather than independent experimental factors. The objective was therefore to evaluate how chuño processing and subsequent thermal treatment influence starch organisation within each system, rather than to attribute effects exclusively to cultivar identity.

Across all samples, chuño processing was associated with changes in amylose content, pasting behaviour, and multiscale structural features. A decrease in amylose content was observed in all cultivars, although the magnitude of this reduction varied among samples. Given the nature of the processing method, which involves repeated freeze–thaw and pressing cycles determined by tuber characteristics, these compositional changes are best interpreted as integrated outcomes of processing history and material-dependent behaviour, rather than the result of a single controlled variable.

Functional analysis indicated reduced peak viscosity and altered pasting stability in chuño-derived starches compared with native starches. These changes are consistent with modifications in hydration behaviour and granule swelling under thermal conditions, although they cannot be attributed to a single mechanistic pathway within the constraints of the present experimental design.

Spectroscopic and diffraction analyses consistently indicated that chuño processing affects starch organisation at different structural levels without modifying its fundamental chemical composition or crystalline polymorph. All samples retained B-type crystallinity, while FT-IR analysis revealed changes in band ratios associated with short- and less-ordered molecular domains. The agreement between FT-IR and XRD results supports the interpretation that chuño processing induces reorganisation of starch structure across hierarchical scales rather than chemical transformation or phase transition.

Thermal treatment produced additional modifications in structural stability, with differences observed among cultivars in the extent of spectral and diffraction changes. However, these differences should be interpreted cautiously, as they likely reflect combined contributions from intrinsic starch properties, prior processing history, and the experimental thermal conditions applied.

Overall, the functional properties of chuño starches, including reduced swelling capacity, lower viscosity development, and altered retrogradation-related parameters, are consistent with a physically modified starch-like behaviour. These observations reflect integrated structural responses rather than single-factor effects.

A key limitation of this study is the inherent coupling between cultivar identity, environmental growth conditions, and processing intensity. Because chuño processing is defined by an endpoint rather than a fixed number of cycles, and because plant material was obtained from distinct agroecological environments, it is not possible within the present design to fully decouple the contributions of genotype, environment, and processing history.

Despite these limitations, the results provide a coherent description of how traditional chuño processing is associated with multiscale modifications in starch structure and functionality. Further studies using controlled cultivation conditions, standardised processing protocols, and expanded molecular characterisation (including fine structure, molecular weight distribution, and enzymatic digestibility) would be required to establish more detailed mechanistic relationships.

## Figures and Tables

**Table 1:** Morphological, culinary and agronomic characteristics of the potato varieties analysed.

Category	Trait	*Condor Imilla* (*S. tuberosum* ssp. *andigena*)	*Luk’i Turno* (*S. × juzepczukii*)	*Dutch Désirée* (*S. tuberosum*)
Morphology	Flower colour	Lilac with reddish-purple tones	Lilac with reddish-purple tones	Pale pink
Morphology	Flower shape	Pentagonal	Pentagonal	Pentagonal
Morphology	Flowering intensity	Moderate	Moderate	High
Morphology	Stem colour	Green with strong pigmentation	Green with weak pigmentation	Green with reddish pigmentation at base
Morphology	Leaf dissection	Intermediate	Weakly dissected	Moderately dissected
Morphology	Tuber shape	Round with deep eyes	Elliptical with medium-depth eyes	Elongated oval
Morphology	Skin colour	Reddish-purple with yellow areas around eyes	Reddish	Pink
Morphology	Flesh colour	Cream	White	Pale yellow
Culinary	Primary use	Boiling	Chuño production	Boiling and frying
Culinary	Glycoalkaloids	Low (non-bitter phenotype)	High (bitter Andean type)	Moderate (mainly in peel tissues)
Agronomy	Growth habit	Decumbent	Decumbent	Semi-erect to decumbent
Agronomy	Vegetative cycle	Late (150–170 days)	Late (150–180 days)	Medium–late (90–120 days)
Agronomy	Productivity	8–10 t ha^−1^	4–6 t ha^−1^	10–20 t ha^−1^
Agronomy	Storage stability	4–6 months	4–6 months	4–6 months
Agronomy	Disease susceptibility	Late blight (*Phytophthora infestans*), early blight (*Alternaria solani*)	Potato wart (*Synchytrium endobioticum*)	Late blight (*Phytophthora infestans*), black scurf (*Rhizoctonia solani*)
Agronomy	Frost tolerance	Moderate	High (down to −4 °C)	Low–moderate
Agronomy	Altitudinal range	3600–3900 m a.s.l.	3500–4000 m a.s.l.	2000–3500 m a.s.l.
Agronomy	Production zones	La Paz, Cochabamba	La Paz, Oruro, Potosí	La Paz, Cochabamba, Santa Cruz, Tarija

Source: Own elaboration based on Ugarte and Iriarte [25].

**Table 2:** Amylose content of starch samples obtained from potato and chuño.

Cultivar	Amylose Content in Potato Starch (%)	Amylose Content in Chuño Starch (%)	Reduction in Amylose Content (%)
*Condor Imilla*	36.89 ± 1.43	33.59 ± 0.9	8.9
*Luk’i Turno*	30.78 ± 0.58	28.67 ± 1.57	6.9
*Dutch Désirée*	32.60 ± 1.07	24.98 ± 0.73	23.4

**Table 3:** Viscoamylograph-derived parameters for potato and chuño starches.

Starch Samples						
	*Condor Imilla* Potato	*Luk’i Turno* Potato	*Dutch Désirée* Potato	*Condor Imilla* Chuño	*Luk’i Turno* Chuño	*Dutch Désirée* Chuño
Onset gelatinisation temperature (°C)	66.75	64.30	64.75	82.60	82.05	72.40
Peak viscosity (BU)	1110.0	457.5	761.0	194.5	200.0	442.5
Temperature at peak viscosity (°C)	85.40	85.00	86.15	85.00	84.95	84.90
Time to reach peak viscosity (min)	12.30	10.78	8.22	12.28	12.30	12.27
Trough viscosity (BU)	1111.00	454.00	669.00	195.00	200.00	441.50
Breakdown viscosity (BU)	−1.0	3.5	92.0	−0.5	0.0	1.0
Final viscosity (BU)	1662.5	560.5	860.0	317.0	240.5	640.5
Setback ratio (%)	−4.79	−3.87	−3.11	−4.70	−4.72	−4.68

**Table 4:** Descriptive statistics of starch granule diameters (µm) for native and chuño starches from three potato varieties: *Condor Imilla*, *Luk’i Turno*, and *Dutch Désirée*.

	Starch Samples					
	*Condor Imilla* Potato	*Luk’i Turno* Potato	*Dutch Désirée* Potato	*Condor Imilla* Chuño	*Luk’i Turno* Chuño	*Dutch Désirée* Chuño
Mean (µm)	31.3	33.9	35.7	29.7	29.7	36.8
Median (µm)	28.8	31.2	33.2	26.6	25.5	34.3
Variance (µm^2^)	194.7	324.5	252.0	270.8	305.2	293.9
Standard deviation (µm)	14.0	18.0	15.9	16.5	17.5	17.1
Minimum (µm)	7.4	7.1	7.6	5.4	4.4	6.1
Maximum (µm)	72.3	88.1	91.7	228.0	85.3	87.9
Range (µm)	64.9	81.0	84.1	222.7	80.9	81.8
Interquartile range (µm)	19.5	28.4	22.5	18.5	26.8	24.0
Skewness (-)	0.6	0.6	0.8	3.9	0.8	0.7

**Table 5:** FT-IR band intensity ratios (A_1047_/A_1022_ of starch samples used to evaluate short-range molecular order. The bands at approximately 1047 cm^−1^ and 1022 cm^−1^ are associated with ordered (crystalline-like) and less ordered (amorphous or disordered) domains, respectively.

A_1047_/A_1022_ Ratio Sample	Potato (RT)	SD	Chuño (RT)	SD	Potato (Thermally Treated)	SD	Chuño (Thermally Treated)	SD
*Condor Imilla*	0.861	0.008	0.640	0.006	0.602	0.005	0.511	0.004
*Luk’i Turno*	0.872	0.003	0.662	0.002	0.681	0.011	0.550	0.005
*Dutch Désirée*	0.890	0.008	0.670	0.008	0.713	0.002	0.563	0.004

RT = Room temperature.

## Data Availability

The original contributions presented in the study are included in the article/Supplementary Material, further inquiries can be directed to the corresponding author.

## Supplementary Materials

The following supporting information can be downloaded at: https://www.mdpi.com/article/10.3390/foods15122180/s1, Figures S1–S3, gelatinisation thermograms of starches from *Condor Imilla*, *Luk’i Turno*, and *Dutch Désirée* potato and corresponding chuño samples; Figures S4–S6, SEM images (100×) of untreated starches; Figures S7–S9, SEM images (100×) of heat-treated starches; Table S1, X-ray diffraction (XRD) peak positions of potato and chuño starches.
