# Supplementary material for: Structural and Functional Effects of Traditional Chuño Processing on Potato Starch (Solanum spp.)
Source: Foods. 2026 Jun 17;15(12):2180. doi: 10.3390/foods15122180 (PMC13298175; doi:10.3390/foods15122180)
Supplement: Supplementary file 1 [file foods-15-02180-s001.zip › foods-4332338-supplementary.pdf]

Supplementary Materials

Structural and Functional Effects of Traditional Chuño Processing on Potato Starch (*Solanum* spp.)

Fabiola Valdivieso [1], José Luis Vila [1], Patricia Mollinedo[1], and Luis Apaza Ticona[2,*]

[1] Chemistry Research Institute, Faculty of Pure and Natural Sciences, Universidad Mayor de San Andrés, Building, 2nd Floor, Laboratory 6, Calle 27, Cota Cota, Campus Universitario, Av. Andrés Bello, La Paz 10077, Bolivia; faavaldivieso@gmail.com (F.V.); jvila@fcpn.edu.bo (J.L.V.); pmollinedo@fcpn.edu.bo (P.M.)

[2] Organic Chemistry Unit, Department of Chemistry in Pharmaceutical Sciences, Faculty of Pharmacy, University Complutense of Madrid, Plza. Ramón y Cajal s/n, 28040 Madrid, Spain (L.A.T.)

* Correspondence: lnapaza@ucm.es

Contents:

- **Figure S1**. Gelatinisation thermogram of *Condor Imilla* potato starch and *Condor Imilla* chuño starch.
- **Figure S2**. Gelatinisation thermogram of starch from *Luk'i Turno* potato and *Luk'i Turno* chuño.
- **Figure S3.** Gelatinisation thermogram of *Dutch Désirée* potato starch and *Dutch Désirée* chuño starch.
- **Figure S4.** SEM images of starches (100×) without heat treatment from *Condor Imilla* potato and *Condor Imilla* chuño.
- **Figure S5.** SEM images of starches (100×) without heat treatment from *Luk'i Turno* potato and *Luk'i Turno* chuño.
- **Figure S6.** SEM images of starches (100×) without heat treatment from *Dutch Désirée* potato and *Dutch Désirée* chuño.
- **Figure S7.** SEM images of starches (100×) with heat treatment from *Condor Imilla* potato and *Condor Imilla* chuño.
- **Figure S8.** SEM images of starches (100×) with heat treatment from *Luk'i Turno* potato and *Luk'i Turno* chuño.
- **Figure S9.** SEM images of starches (100×) with heat treatment from *Dutch Désirée* potato and *Dutch Désirée* chuño.
- **Table S1.** X-ray Diffraction (XRD) peaks of potato and chuño varieties.

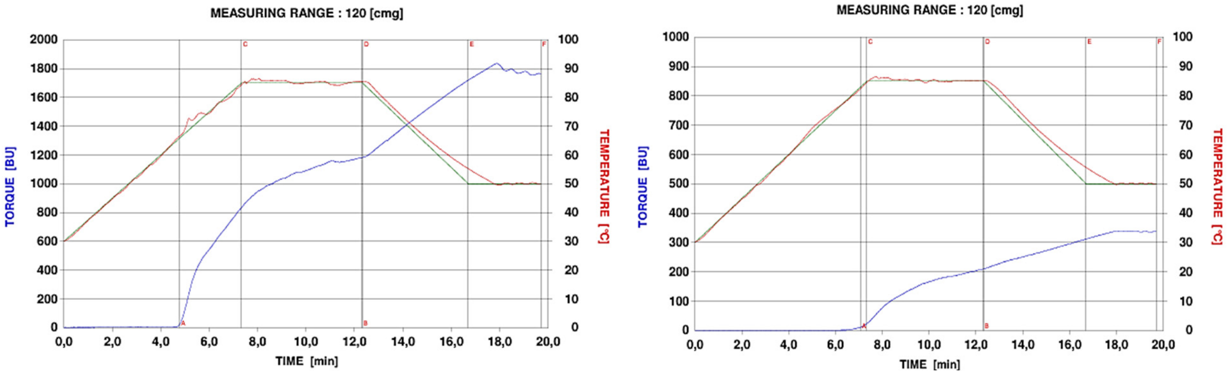

- **Figure S1**. Gelatinisation thermogram of *Condor Imilla* potato starch and *Condor Imilla* chuño starch.

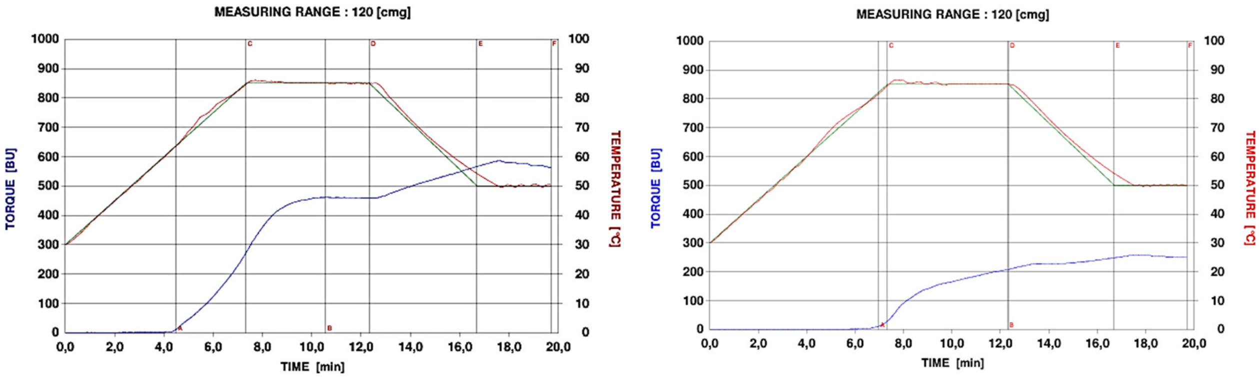

- **Figure S2**. Gelatinisation thermogram of starch from *Luk'i Turno* potato and *Luk'i Turno* chuño.

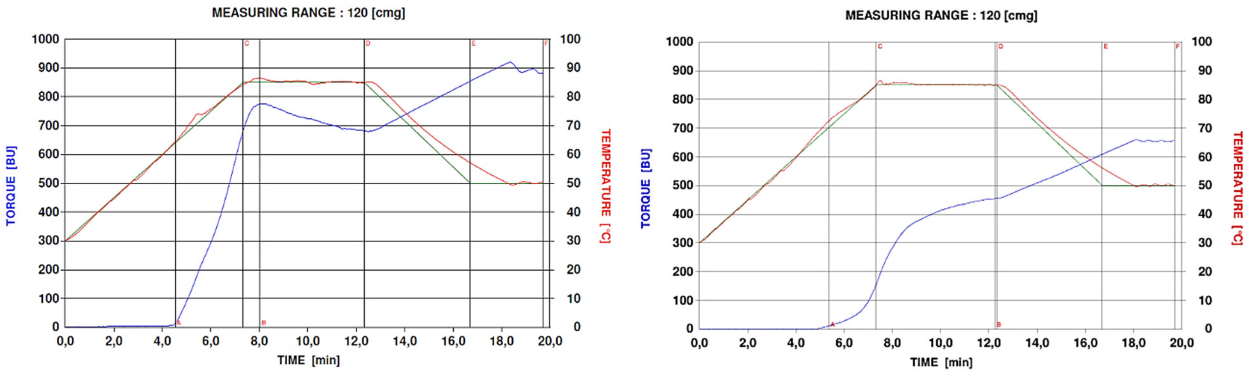

- **Figure S3.** Gelatinisation thermogram of *Dutch Désirée* potato starch and *Dutch Désirée* chuño starch.

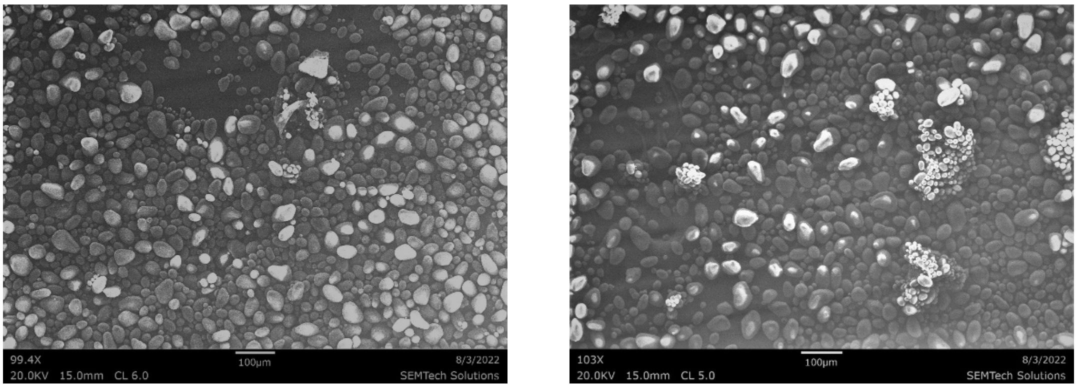

- **Figure S4.** SEM images of starches (100×) without heat treatment from *Condor Imilla* potato and *Condor Imilla* chuño.

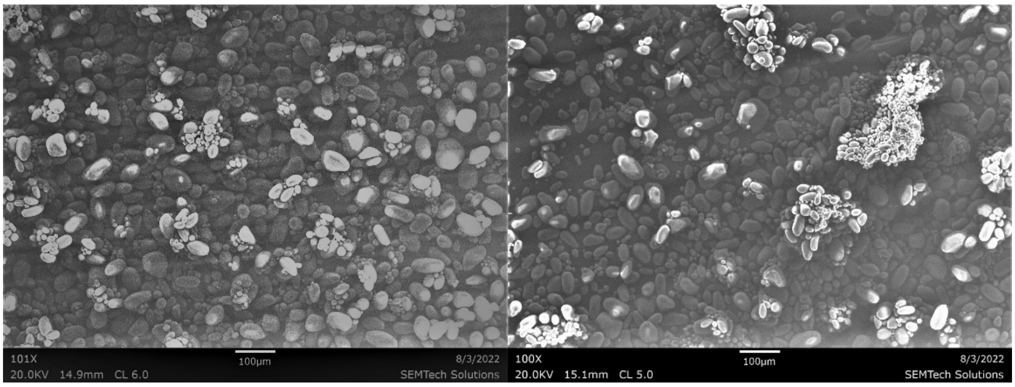

- **Figure S5.** SEM images of starches (100×) without heat treatment from *Luk'i Turno* potato and *Luk'i Turno* chuño.

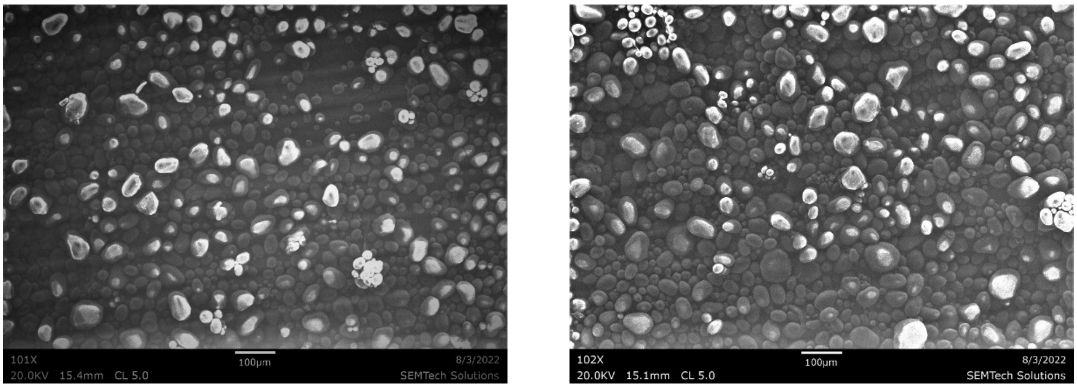

- **Figure S6.** SEM images of starches (100×) without heat treatment from *Dutch Désirée* potato and *Dutch Désirée* chuño.

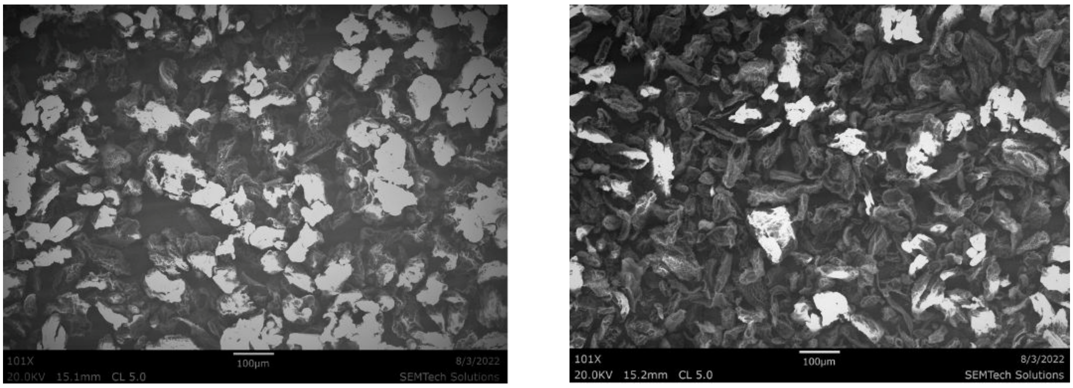

- **Figure S7.** SEM images of starches (100×) with heat treatment from *Condor Imilla* potato and *Condor Imilla* chuño.

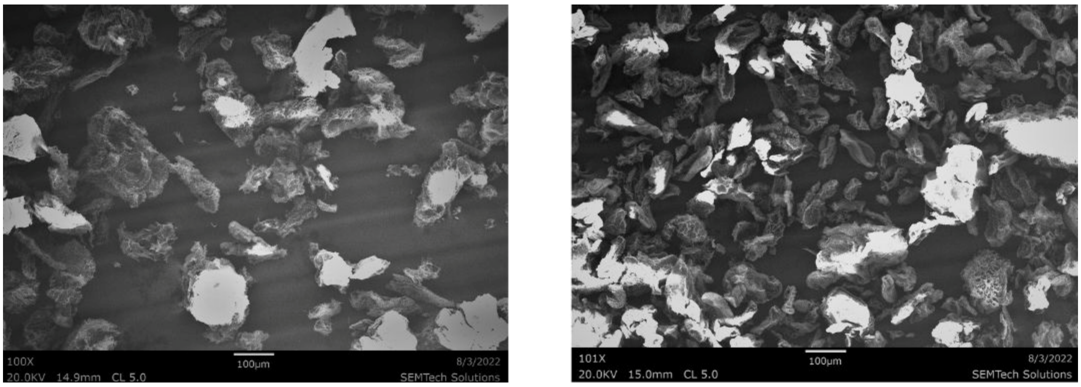

- **Figure S8.** SEM images of starches (100×) with heat treatment from *Luk'i Turno* potato and *Luk'i Turno* chuño.

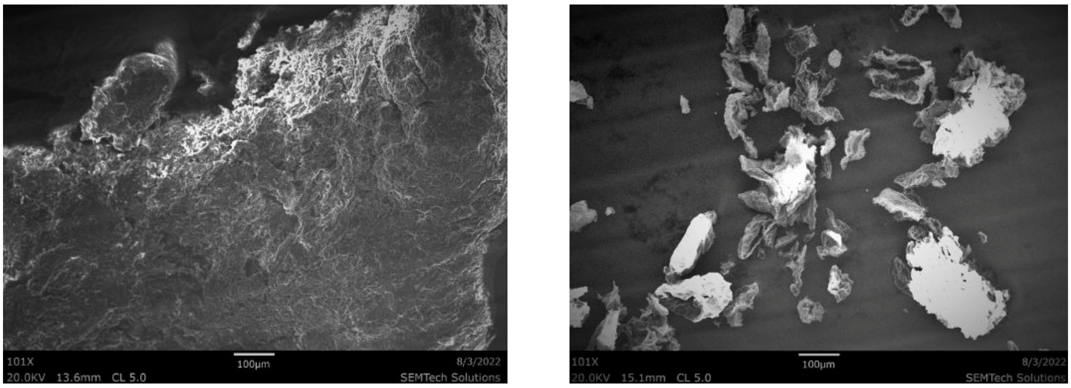

- **Figure S9.** SEM images of starches (100×) with heat treatment from *Dutch Désirée* potato and *Dutch Désirée* chuño.

- **Table S1.** X-ray Diffraction (XRD) peaks of potato and chuño varieties.

**X-ray Diffraction Spectrum Peaks (2θ (°))**						
**Theoretical Crystallinity – Type B**	***Condor Imilla* Potato**	***Condor Imilla* Chuño**	***Luk'i Turno* Potato**	***Luk'i Turno* Chuño**	***Dutch Désirée* Potato**	***Dutch Désirée* Chuño**
5.51	5.60422	5.54	5.6	5.6	5.659	5.62
10.01	10.07	10.2	10.01	10.01	10.01	10.01
11.02	11.4	11.42	11.02	11.42	11.3	11.3
13.85	13.84	14	13.9	13.8	13.85	13.8
14.6	15	15	15	14.96	15	15
16.85	17.06	16.99	17.11	17.1	17.15	17.05
		19.6		19.88	19.74	19.74
22.3	22.2	22.2	22.4	22.4	22.28	22.3441
23.71	23.9	23.95	24.11	24.08	24.11	24.11
26.16	26.01	26.16	26.19	26.16	26.4	26.16
30.61	30.21	30.61	30.61	30.61	30.64	30.61
33.84	33.84	34	33.84	33.84	34.24	33.84

(shaded) Peak not associated with B-type crystallinity
